# Supplementary material for: Intralocus conflicts associated with a supergene
Source: Nat Commun. 2022 Mar 16;13:1384. doi: 10.1038/s41467-022-29033-w (PMC8927407; doi:10.1038/s41467-022-29033-w)
Supplement: Supplementary file 1 — Supplementary Information [file 41467_2022_29033_MOESM1_ESM.docx]

Supplementary Information

**Supplementary Methods**

*Hormone analysis -* To determine hormone concentrations of testosterone, androstenedione and progesterone in yolk we used a modification ^1,2^ of the method described by Schwabl (1993). Because androstenedione and progesterone are eluted in the same fraction, we ran column chromatography for each sample twice: the first set of samples was analyzed ^4^ for androstenedione and testosterone, and the second set of samples was analyzed for progesterone, only. Each yolk sample (200 mg yolk-water homogenate) was added with 500 µl of milliQ water and 10 µl of the respective radioactive tracers (3H-labelled androstenedione and testosterone for the first set of extractions, and progesterone for the second set of extractions; Perkin-Elmer) to estimate the hormone recovery during the extraction. After four hours of equilibration, 4ml diethylether was added to the samples. After overnight equilibration, the organic phase was separated from the aqueous phase by plunging the extraction tubes into a methanol - dry ice bath and decanting the diethylether phase. After a second extraction with 2ml of dichloromethane the samples were dried under a stream of nitrogen at 39°C. Then, extracts were resuspended with 100% ethanol and kept at -20°C overnight to precipitate proteins and lipids. After centrifugation, the ethanol-phase was transferred into new tubes and dried under a stream of nitrogen at 39°C. Then, two times 250µl of 2% ethylacetate in isooctane was added to each tube, vortexed and then transferred to the top of chromatography columns containing a mixture of diatomaceous earth : propylene glycol : ethylene glycol (6:1.5:1.5). Steroid hormones were separated on the basis of their polarity by eluting columns with 4 ml portions of increasing concentrations of ethyl acetate in isooctane (2% for androstenedione and progesterone, 10% for dihydrotestosterone (not measured) and 25% for testosterone). Collected fractions were dried at 39°C and then re-dissolved in 300µl phosphate buffered saline with 1% gelatine (PBSG) and left overnight at 4°C to equilibrate. Aliquots (80 µl) of each fraction and sample were transferred to scintillation vials, mixed with 4ml scintillation fluid (Packard Ultima Gold) and counted to an accuracy of 2-3% in a Beckman LS 6000-counter to estimate individual extraction recoveries. Recoveries were 69.5% ± 6.2% for androstenedione, 51.2% ± 10.2% for progesterone, and 48.1% ± 6.5% for testosterone; N=128 eggs. The remainder was stored at -40°C until RIA was conducted.

Standard curves were set up by serial dilution of stock standard solutions with a concentration range of 0.4-200pg for androstenedione and testosterone, and 1.0-500pg for progesterone. Dilutions of the respective antisera (1/200 for each hormone; all antibodies from Esoterix Endocrinology, Calabasas Hill, CA) were added to the standard curve, controls and to duplicates of the respective sample fractions (2 x 100µl). After 30 min 13500 dpm of 3H-labelled androstenedione or testosterone, and 5000 dpm for progesterone, were added to all tubes of the respective assay and incubated for 20 hours at 4°C. Antibody-bound and free fractions of the respective hormone were separated at 4°C by adding 0.5 ml dextran-coated charcoal. After 14 min incubation with charcoal samples were spun (3600g, 10min, 4°C) and supernatants decanted into scintillation vials at 4°C. After adding 4 ml scintillation liquid (Packard Ultima Gold) vials were counted. The standard curve and the sample concentrations were calculated with Immunofit 3.0 (Beckman Inc. Fullerton, CA), using a four-parameter logistic curve fit. In total we conducted 5 assays per hormone. The lower detection limit of the standard curves was determined as the first value outside the 95% confidence intervals for the zero standard (Bmax) and ranged from 7.2 – 9.3pg/ml for androstenedione, 20.9 – 38.4 pg/ml for progesterone, and was 2.9 – 5 pg/ml for testosterone. All samples were within the detectable range. The intra-assay variation based on standard samples containing chicken plasma that were extracted with the yolk samples was 6.8% ± 2.9% for androstenedione, 9.8% ± 6.0% for progesterone, and 16.9% ± 8.9% for testosterone. The inter-assay variation was 11.7% for androstenedione, 12.0% for progesterone, and 6.0% for testosterone.

*Parentage assignment -* We used 21 polymorphic microsatellite markers and two sex chromosome linked marker (Supplementary Table 6). Microsatellites were amplified with the Qiagen Type-it Microsatellite PCR Kit (Qiagen) combining seven or eight primer pairs (Supplementary Table 6) in each multiplexed PCR. The forward primer of each pair was fluorescently labelled with dyes 6-FAM, VIC, PET or NED (Dye Set G5; Thermo-Fisher Scientific). Differences in amplification efficiency and dye strength of the primers were accommodated by adapting the primer concentrations in these mixes (Supplementary Table 6). Each 10μl multiplex PCR contained 20 – 80ng DNA, 5μl of the 2x Type-it Microsatellite PCR Master Mix and 1μl of one of the primer mixes. Cycling conditions were: 5min initial denaturation at 95˚C; 28 cycles of 30s denaturation at 94˚C, 90s annealing at 50°C (mix 40) or 53°C (mix 41 and 42), and 1min extension at 72˚C; followed by a 30min completing final extension at 60˚C. Samples from eggs with low DNA concentrations were boosted with up to 40 cycles.

For fragment length analysis, an aliquot of 1.5μl of each PCR product was added to 13μl formamide containing the GeneScan 500 LIZ Size Standard, then heat denatured and resolved in POP7 polymer on an ABI 3130xl Genetic Analyzer (ThermoFisher Scientific). Samples with Phil2 alleles >500bp were reloaded with the bigger size standard GeneScan 1200 LIZ. For allele assignment, we used the software GeneMapper 4.0.

Since the pool of potential parents was limited and all potential parents were genotyped, parentage was assigned by exclusion. This was done by counting the mismatches between the sample genotype and all pairs of potential parents. Parentage was assigned to the best matching pair that usually showed zero mismatches (a few cases of mismatches could be traced to genotyping errors in samples from dead embryos due to DNA degradation).

**SupplementaryTables**

**Supplementary Table 1**. Posterior distributions of parameter estimates for maternal morph, orthogonal linear and quadratic term of maternal age and sampling year on laying rate (eggs per female per year). Given are the mean, 95% CrI, and the posterior probability of the hypothesis that the parameter is smaller than zero. The residual standard deviation was 0.95 (0.74; 1.29). P values in bold are comparable to a significant effect (P<0.05) according to frequentist statistics.

| **Parameter** | **Mean** | **95% CrI** | **P (β<0)** |
| --- | --- | --- | --- |
| Intercept | 2.42 | 1.76; 3.08 | **<0.001** |
| Satellite mother | -0.25 | -1.01; 0.49 | 0.74 |
| Faeder mother | -1.48 | -2.69; -0.24 | **0.99** |
| age linear | -1.08 | -3.01; 0.87 | 0.87 |
| age quadratic | -3.42 | -5.62; -1.26 | **>0.99** |
| year 2018 | -0.92 | -1.74; -0.1 | **0.99** |
| year 2019 | 0.72 | -0.16; 1.60 | 0.05 |

**Supplementary Table 2.** Posterior distributions of parameter estimates for female morph on egg mass (in g) with female ID and sampling year included as random factors in the model. The intercept provides the estimate for Independent females. Given are the mean, 95% CrI, and the posterior probability of the hypothesis that the parameter is smaller than zero. The between-female standard deviation was 0.98, the between-sampling year standard deviation was 0.18 and the residual standard deviation was 0.95. P values in bold are comparable to a significant effect (P<0.05) according to frequentist statistics.

| **Parameter** | **Mean** | **95% CrI** | **P (β<0)** |
| --- | --- | --- | --- |
| Intercept | 18.81 | 18.47; 19.15 | **<0.01** |
| Satellite mother | -0.10 | -0.71; 0.51 | 0.62 |
| Faeder mother | -1.53 | -2.46; -0.59 | **>0.99** |

**Supplementary Table 3.** Posterior distributions of parameter estimates for female morph on deviation of the observed egg mass from the expected egg mass according to female body mass. For expected egg mass calculations we used the formula egg mass = 0.613*female body mass^0.726^ ^5^. We included female ID and sampling year as random factors in the model. The intercept provides the estimate for Independent females. Given are the mean, 95% CrI, and the posterior probability of the hypothesis that the parameter is smaller than zero. The between-female standard deviation was 0.11, the between-sampling year standard deviation was 0.01 and the residual standard deviation was 0.06. P values in bold are comparable to a significant effect (P<0.05) according to frequentist statistics.

| **Parameter** | **Mean** | **95% CrI** | **P (β<0)** |
| --- | --- | --- | --- |
| Intercept | 0.06 | 0.03; 0.09 | **<0.01** |
| Satellite mother | 0.01 | -0.06; 0.08 | 0.41 |
| Faeder mother | 0.20 | 0.10; 0.31 | **<0.01** |

**Supplementary Table 4.** Posterior distributions of parameter estimates for maternal morph, offspring sex, offspring morph and egg mass on hatching and fledging probability with mother ID and sampling year included as random factors in the models. The intercept provides the estimate for Independent mothers for a female, Independent offspring and a mean egg mass. Given are mean values, 95% CrI, and the posterior probability of the hypothesis that the parameter is smaller than zero. For hatching success, the between-mother ID standard deviation was 0.84 and the between-sampling year standard deviation was 0.10. For fledging success, the between-mother ID standard deviation was 0.51 and the between-sampling year standard deviation was <0.001. P values in bold are comparable to a significant effect (P<0.05) according to frequentist statistics.

| **Parameter** | **Hatching success**  **(N_Offspring_=400, N_Mothers_=87)** | | | **Fledging success**  **(N_Offspring_=165, N_Mothers_=62)** | | |
| --- | --- | --- | --- | --- | --- | --- |
|  | **Mean** | **95% CrI** | **P (β<0)** | **Mean** | **95% CrI** | **P (β<0)** |
| Intercept | -0.49 | -0.94; -0.04 | **0.98** | 0.85 | 0.18; 1.51 | **0.01** |
| Satellite mother | -0.44 | -1.32; 0.43 | 0.84 | 0.32 | -0.93; 1.58 | 0.31 |
| Faeder mother | -1.60 | -3.05; -0.14 | **0.98** | 0.20 | -2.19; 2.58 | 0.43 |
| male offspring | 0.31 | -0.13; 0.74 | 0.09 | -0.50 | -1.20; 0.23 | 0.91 |
| Satellite offspring | 0.22 | -0.31; 0.74 | 0.21 | 0.23 | -0.60; 1.05 | 0.29 |
| Faeder offspring | 0.19 | -0.45; 0.84 | 0.29 | 0.24 | -0.77; 1.26 | 0.32 |
| egg mass | 0.04 | -0.22; 0.31 | 0.38 | 0.50 | 0.12; 0.89 | **<0.01** |

**Supplementary Table 5.** Means and 95% CrI of the posterior distributions for hatching and fledging probabilities according to the maternal morph, the offspring sex, the offspring morph and the overall mean egg mass. Mother ID and sampling year were included as random factors in the models.

| **mother** | **offspring** | | **hatching probability** | | **fledging probability** | |
| --- | --- | --- | --- | --- | --- | --- |
|  |  |  | **mean** | **95% CrI** | **mean** | **95% CrI** |
| Ind. | female | Ind. | 0.38 | (0.28; 0.49) | 0.70 | (0.55; 0.82) |
|  |  | Sat. | 0.43 | (0.30; 0.57) | 0.75 | (0.57; 0.87) |
|  |  | Faed. | 0.43 | (0.28; 0.59) | 0.75 | (0.51; 0.89) |
|  | male | Ind. | 0.45 | (0.35; 0.56) | 0.59 | (0.45; 0.72) |
|  |  | Sat. | 0.51 | (0.37; 0.65) | 0.64 | (0.44; 0.80) |
|  |  | Faed. | 0.50 | (0.34; 0.66) | 0.65 | (0.42; 0.82) |
| Sat. | female | Ind. | 0.28 | (0.14; 0.49) | 0.76 | (0.46; 0.93) |
|  |  | Sat. | 0.33 | (0.18; 0.53) | 0.80 | (0.54; 0.93) |
|  |  | Faed. | 0.32 | (0.14; 0.58) | 0.81 | (0.45; 0.95) |
|  | male | Ind. | 0.35 | (0.18; 0.56) | 0.66 | (0.36; 0.88) |
|  |  | Sat. | 0.40 | (0.22; 0.61) | 0.71 | (0.42; 0.90) |
|  |  | Faed. | 0.39 | (0.18; 0.65) | 0.72 | (0.36; 0.92) |
| Faed. | female | Ind. | 0.11 | (0.03; 0.35) | 0.74 | (0.19; 0.97) |
|  |  | Sat. | 0.13 | (0.03; 0.42) | 0.78 | (0.23; 0.98) |
|  |  | Faed. | 0.13 | (0.03; 0.38) | 0.79 | (0.27; 0.97) |
|  | male | Ind. | 0.14 | (0.04; 0.43) | 0.63 | (0.13; 0.95) |
|  |  | Sat. | 0.17 | (0.04; 0.49) | 0.69 | (0.15; 0.96) |
|  |  | Faed. | 0.17 | (0.05; 0.45) | 0.69 | (0.20; 0.95) |

**Supplementary Table 6.** Characterization of microsatellite loci for parentage assignment based on 782 genotyped ruffs. Primer sequences include information on fluorescence labels used. C indicates the primer concentration in multiplex primer mix, Ta the annealing temperature, nA the number of alleles, H(obs) the observed heterozygosity, and H(exp) the expected heterozygosity.

| **Locus** | **Accession no.** | **published in** | **designed for**  **(original species)** | **Primer sequences (5’ - 3’)** | **Multiplex Mix** | **C**  **(μM)** | **Ta**  **(°C)** | **Size range**  **(bp)** | **nA** | **H(obs)** | **H(exp)** |
| --- | --- | --- | --- | --- | --- | --- | --- | --- | --- | --- | --- |
| **Cme9** | DQ825682 | Carter and Kempenaers (2007) | pectoral sandpiper  (*Calidris melanotos*) | 6FAM-CCAACAAGAAGGGAAAACTGC ACAGAGCCTTTTGCCCACAG | 40 | 0,14 | 50 | 130-154 | 8 | 0.778 | 0.745 |
| **Ruff1 ††** | AF473568 | Thuman et al. (2002) | ruff  (*Philomachus pugnax*) | 6FAM-TTTCCAAGAGACCAGCAATAAG GATTGCTTTGGCTGGAGATG | 40 | 0,28 | 50 | 181-197 | 5 | 0.631 | 0.621 |
| **Ppu47** | HE616957 | Farrell et al. (2012) | ruff  (*Philomachus pugnax*) | 6FAM-TGCAGCTTTAATTGCAACAGCTAATC AGCGCTCAGGTCTGAATGAGTTC | 40 | 0,22 | 50 | 288-292 | 3 | 0.534 | 0.536 |
| **P2P8** ***** | AF006659-62 | Griffiths et al. (1998) | diverse | 6FAM-CTCCCAAGGATGAGRAAYTG TCTGCATCGCTAAATCCTTT | 40 | 0,42 | 50 | 362 (Z)  392 (W) ***** | 2 ***** | - | - |
| **Ruff12** | AF473575 | Thuman et al. (2002) | ruff  (*Philomachus pugnax*) | VIC-ATTCCAAACAAATTGCCTAAGG CGCTGGAAAAGGTGTTTAGGT | 40 | 0,3 | 50 | 216-264 | 12 | 0.864 | 0.868 |
| **Ruff8 †** | AF473572 | Thuman et al. (2002) | ruff  (*Philomachus pugnax*) | PET-ATCTTGCAGGAATCAAAAATGTG TGGCTGTCATTTACTCTGTGTTG | 40 | 0,26 | 50 | 100-156 | 11 | 0.918 | 0.841 |
| **Ruff50** | AF473576 | Thuman et al. (2002) | ruff  (*Philomachus pugnax*) | NED-GAACAGAACAGGACACAAGCTG TGCAACAGAAACCCATATAAGC | 40 | 0,16 | 50 | 187-223 | 7 | 0.611 | 0.576 |
| **Ppu20** | HE616930 | Farrell et al. (2012) | ruff  (*Philomachus pugnax*) | 6FAM-TCCTGTCCTGTCCTTGGAAC GCGGTATTTCTGGCCTAGC | 41 | 0,1 | 53 | 241-249 | 5 | 0.581 | 0.545 |
| **Ppu22 ††** | HE616932 | Farrell et al. (2012) | ruff  (*Philomachus pugnax*) | 6FAM-TGAATGCATGAATTAGGTAGTGG GGGAAACATCATGCAACAAC | 41 | 0,14 | 53 | 264-302 | 8 | 0.866 | 0.854 |
| **Ppu24** | HE616934 | Farrell et al. (2012) | ruff  (*Philomachus pugnax*) | PET-GGAAACCTTCCCATCAACAG GAAGGGATGCATGGTTGG | 41 | 0,16 | 53 | 127-163 | 9 | 0.837 | 0.833 |
| **Ppu25** | HE616935 | Farrell et al. (2012) | ruff  (*Philomachus pugnax*) | 6FAM-GATCCAGACTGCCTAAACAGC GCATCACAAATGCAACTTCAG | 41 | 0,24 | 53 | 332-352 | 8 | 0.863 | 0.841 |
| **Ppu28** | HE616938 | Farrell et al. (2012) | ruff  (*Philomachus pugnax*) | 6FAM-CCTGAACCATTAGTTTACTTGCTG GCACCAGAACTGCCACATAG | 41 | 0,1 | 53 | 185-197 | 4 | 0.631 | 0.620 |
| **Cme6** | DQ825679 | Carter and Kempenaers (2007) | pectoral sandpiper  (*Calidris melanotos*) | VIC-GAGCAGTGCTGCTGACTTTG GCTCTTCCTTGCTTTCATTTC | 41 | 1,4 | 53 | 189-207 | 10 | 0.863 | 0.858 |
| **Ppu48** | HE616958 | Farrell et al. (2012) | ruff  (*Philomachus pugnax*) | PET-TGCAGCATTCTTCGCAGCTA AACACACTGAGCGTCGTTTTATCA | 41 | 0,24 | 53 | 226-234 | 5 | 0.422 | 0.430 |
| **Cme2** | DQ825675 | Carter and Kempenaers (2007) | pectoral sandpiper  (*Calidris melanotos*) | NED-GCTTTCAAGAACATCTGAATCCT TTAAAAGGGACCGAGTGTCCT | 41 | 1,2 | 53 | 156-168 | 6 | 0.749 | 0.731 |
| **Phil2** ¥ | - | Verkuil et al. (2012) | ruff  (*Philomachus pugnax*) | 6FAM-TGAAGGTTTGTCACTGCAAGA GCTTAAAGATTACTTGGGGGAG | 42 | 0,36 | 53 | 207-750 ¥ | 30 | 0.904 | 0.919 |
| **Ppu19** | HE616929 | Farrell et al. (2012) | ruff  (*Philomachus pugnax*) | 6FAM-TAACCCACGAGTGGCTCTG GCTACTGGGTGCTGTTACTTCC | 42 | 0,16 | 53 | 145-161 | 7 | 0.757 | 0.774 |
| **Z37B *** | DV945670 / AC186343 | Dawson et al. (2015) | diverse | 6FAM-AACTGGTTGTAGGTATAGTGCAATTATG GATTACAAAGCCAATATGGATGC | 42 | 0,16 | 53 | 91 (W)  93 (Z) ***** | 2 ***** | - | - |
| **Ruff6** | AF473571 | Thuman et al. (2002) | ruff  (*Philomachus pugnax*) | VIC-GAAACCTTCCCATCAACAGAGTA CAGAATGAAATATAGTTGCAGCAC | 42 | 0,22 | 53 | 152-188 | 9 | 0.837 | 0.834 |
| **Ppu9** | HE616919 | Farrell et al. (2012) | ruff  (*Philomachus pugnax*) | PET-TCTTTATGATGCTATTTGAGGGTTTGG AATGCCACTGCACCAGAAGTAGTC | 42 | 0,26 | 53 | 214-230 | 7 | 0.678 | 0.698 |
| **Tgu06 §** | CK307697 | Slate et al. (2007) | zebra finch (*Taeniopygia guttata)* and chicken (*Gallus gallus)* | PET-CGAGTAGCGTATTTGTAGCGA AGGAGCGGTGATTGTTCAGT | 42 | 1,32 | 53 | 186-194 | 6 | 0.541 | 0.542 |
| **Ppu31** | HE616941 | Farrell et al. (2012) | ruff  (*Philomachus pugnax*) | NED-TGATTCTTATTAGGATTATTTGATGC TGAGGACTGTGGTTTAAGAGC | 42 | 0,17 | 53 | 321-327 | 4 | 0.344 | 0.335 |
| **Ppu56** | HE616966 | Farrell et al. (2012) | ruff  (*Philomachus pugnax*) | NED-CCTCTGGCAAATACTCAATGC CACTGGAAAGGTCAGGAAGC | 42 | 0,14 | 53 | 139-149 | 3 | 0.481 | 0.482 |

¥ Samples with Phil2 alleles >500bp were reloaded with the bigger size standard GeneScan 1200 LIZ for precise sizing.

† Z-chromosomal marker (hemizygous in females).

* For the two sex chromosomal markers the fragment sizes from W- and Z-chromosome are shown.

§ The name of loci Tgu06 was not published in the original but by Klein et al. (2009) and is not unique.

†† Markers containing alleles with different amplification efficiencies resulting in erratic heterozygote peak height ratios.

**Supplementary Table 7.** Numbers of hatched and unhatched chicks from fertile eggs laid by Faeder females.

| mother ID | hatched | unhatched |
| --- | --- | --- |
| 808 | 0 | 1 |
| 847 | 1 | 1 |
| 829 | 1 | 3 |
| 935 | 1 | 8 |
| 960 | 1 | 10 |
| 1060 | 0 | 1 |

**Supplementary Figures**


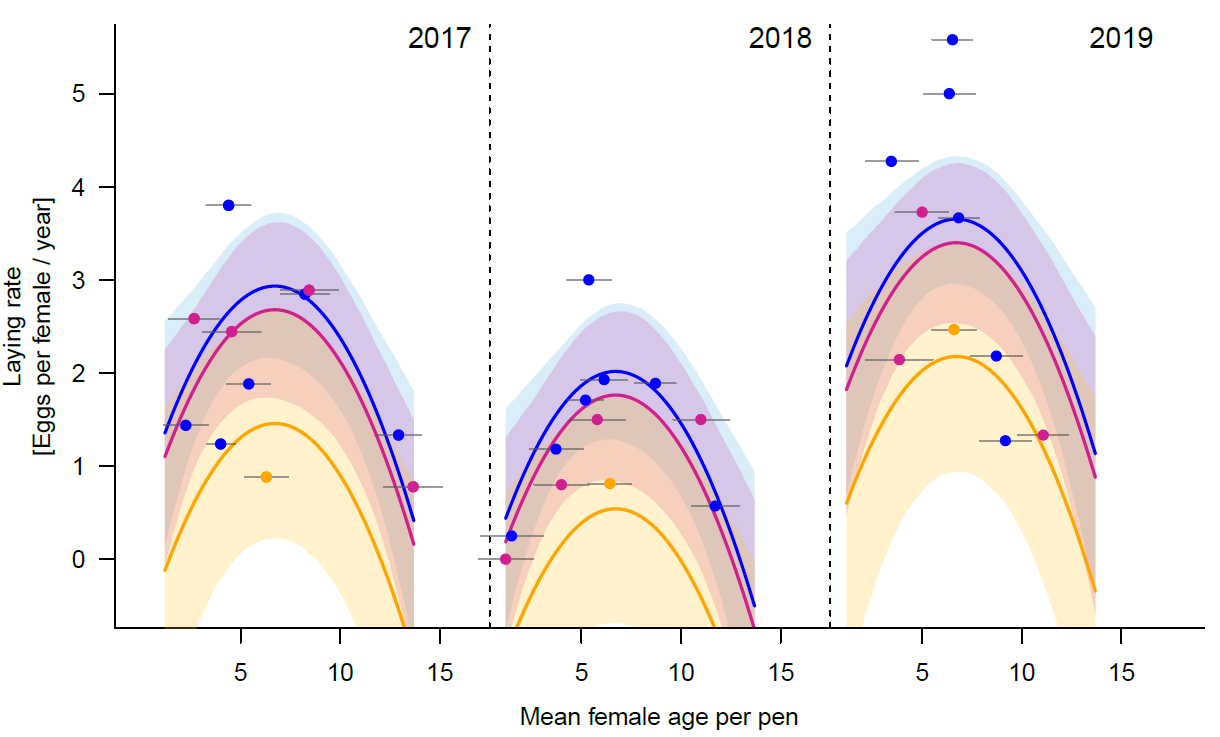


**Supplementary Figure 1 | Annual laying rates of the three female morphs in relation to mean female age per pen for three sampling years.** Lines and their surrounding shading refer to means and 95% CrI of the posterior distribution for Independents (N=19 pens) in blue, Satellites (N=11 pens) in purple and Faeders (N=3 pens) in orange. Coloured circles represent observed mean laying rates from breeding pens. Grey bars indicate the 95% CrI for female mean age in each pen. The probabilities of Faeder females laying fewer eggs than Independent or Satellite females were 99% and 96%, respectively. The probability of Satellite females laying fewer eggs than Independents was 74%. For full model details see Supplementary Table 1. Source data are provided as a Source Data file.


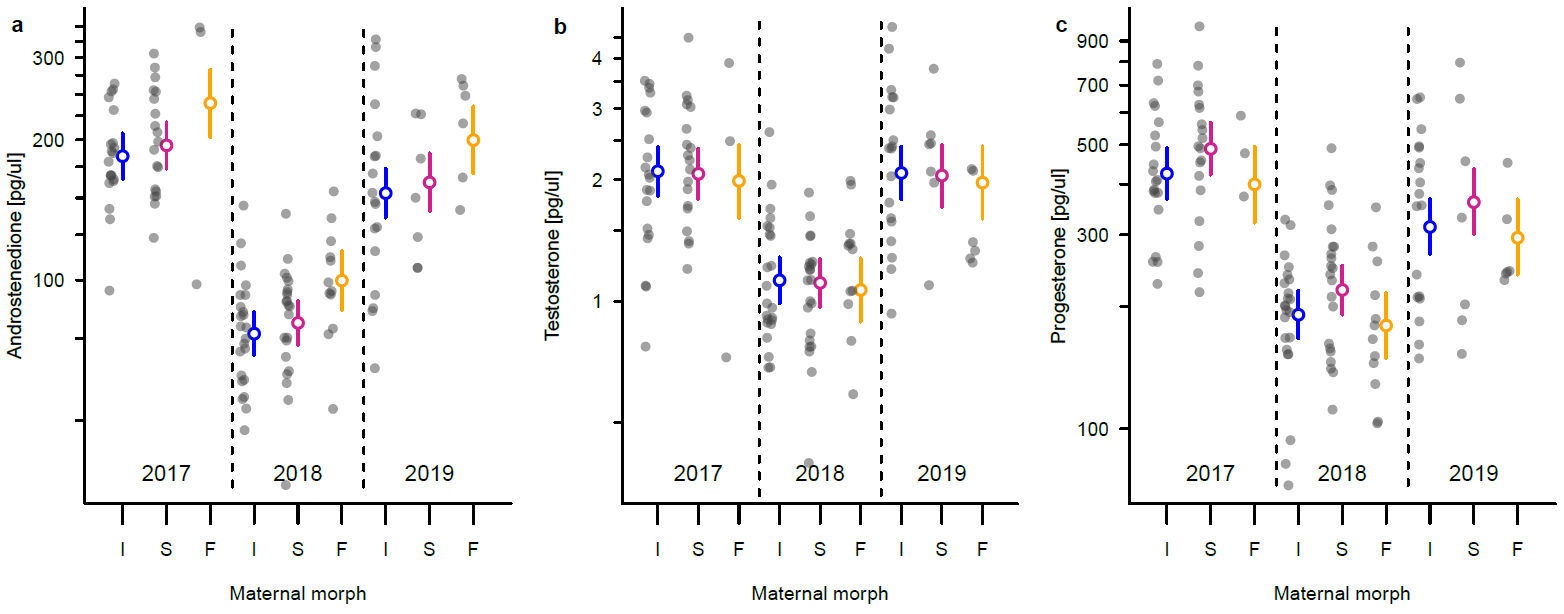


**Supplementary Figure 2** **| Variation in yolk steroid concentrations in eggs produced by different morphs.** Mean and 95% CrI for Independent (I, blue; N=61 eggs), Satellite (S, purple; N=48 eggs) and Faeder females (F, orange; N=21 eggs) across three sampling years (2017, 2018 and 2019). Y-axis is in a logarithmic scale. **a** Yolk androstenedione concentrations. The probabilities of Faeder females laying eggs with higher androstenedione concentrations than Independent or Faeder females were both higher than 0.99. **b** Yolk testosterone concentrations. No clear differences among morphs. **c** Yolk progesterone concentrations. No clear differences among morphs. Source data are provided as a Source Data file.


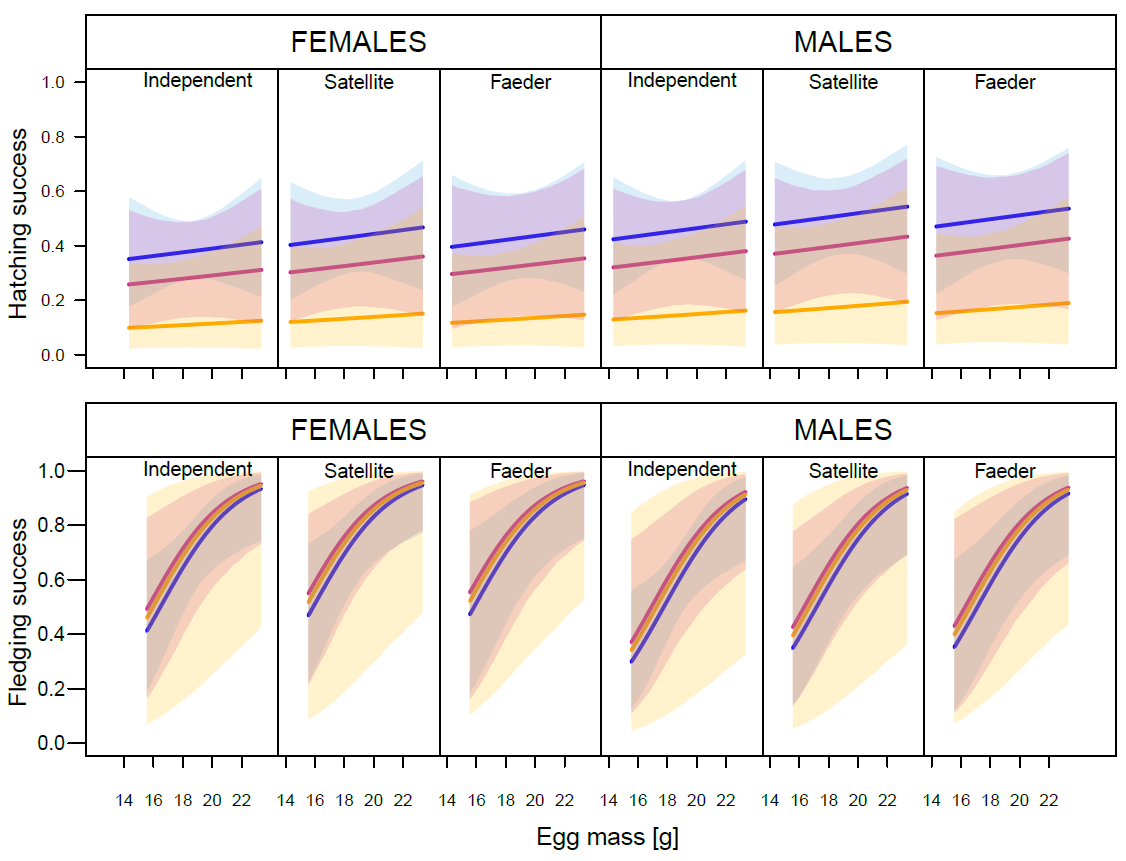


**Supplementary Figure 3** | **Hatching and fledging success in ruff offspring according to egg mass, maternal morph, offspring sex and offspring morph.** Hatching and fledging probabilities (mean and 95% CrI) for offspring of Independent (blue), Satellite (purple) and Faeder (orange) mothers according to the sex and the morph (Independent, Satellite or Faeder) of their offspring. Sample sizes were 400 eggs from 87 mothers for hatching success and 165 chicks from 62 mothers for fledging success. Source data are provided as a Source Data file.


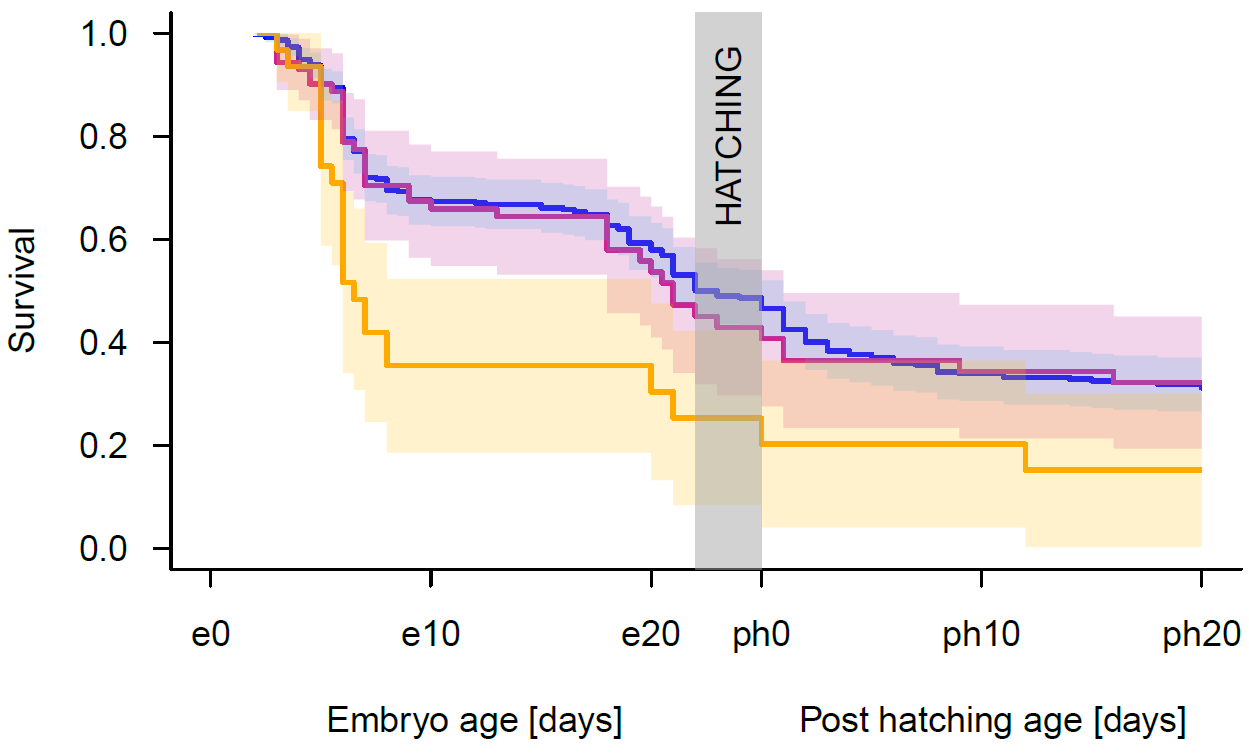


**Supplementary Figure 4** | **Survival according to maternal morph**. Survival probability (mean ± 95% CrI) of offspring from Independent (blue, N_Offspring_=374, N_females_=67), Satellite (purple, N_Offspring_=66, N_females_=16) and Faeder females (orange, N_Offspring_=32, N_females_=6) from an age of 2 days of embryonic development (e) until an age of 20 days post hatching (ph). Chicks hatched between 22-24 days (grey bar). Source data are provided as a Source Data file.


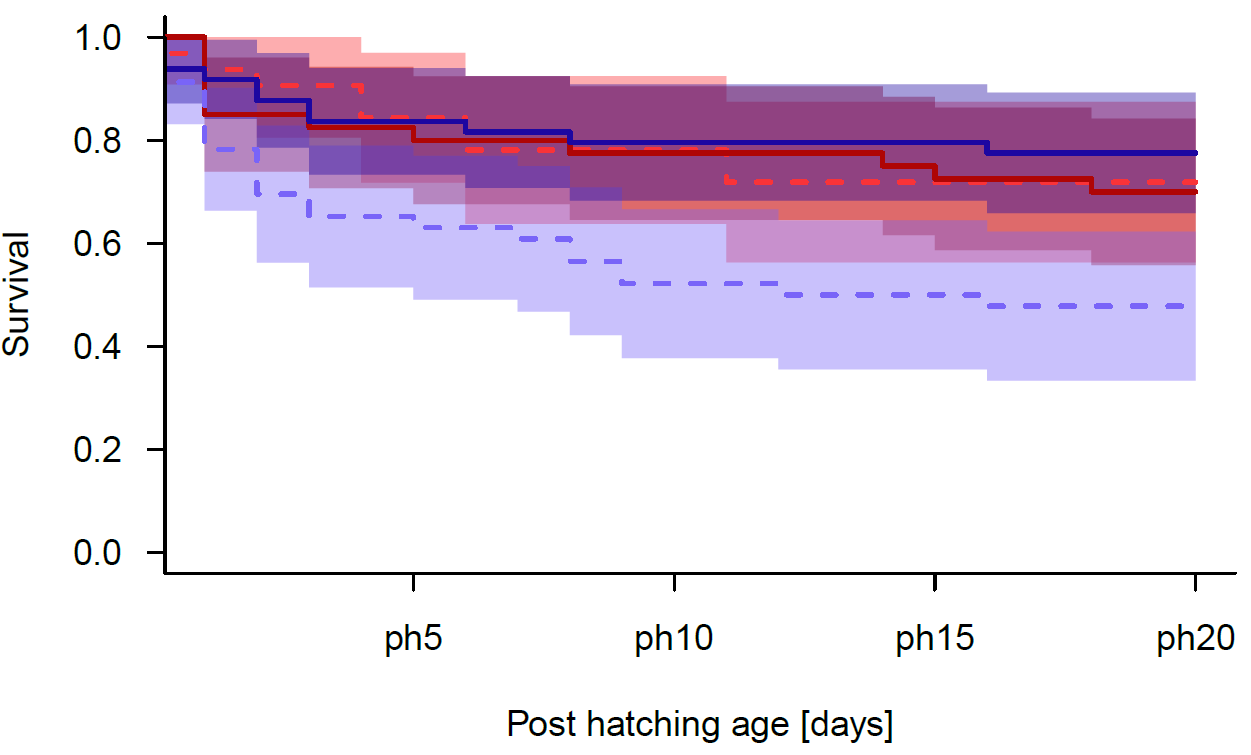


**Supplementary Figure 5 |** **Offspring survival with respect to category of egg mass and sex**. Survival probability (mean ± 95% CrI) of males (blue, N=247) and females (red, N=225) hatched from eggs that were heavier (solid line) or lighter (broken line) than the overall mean egg mass (18.68g). Source data are provided as a Source Data file.


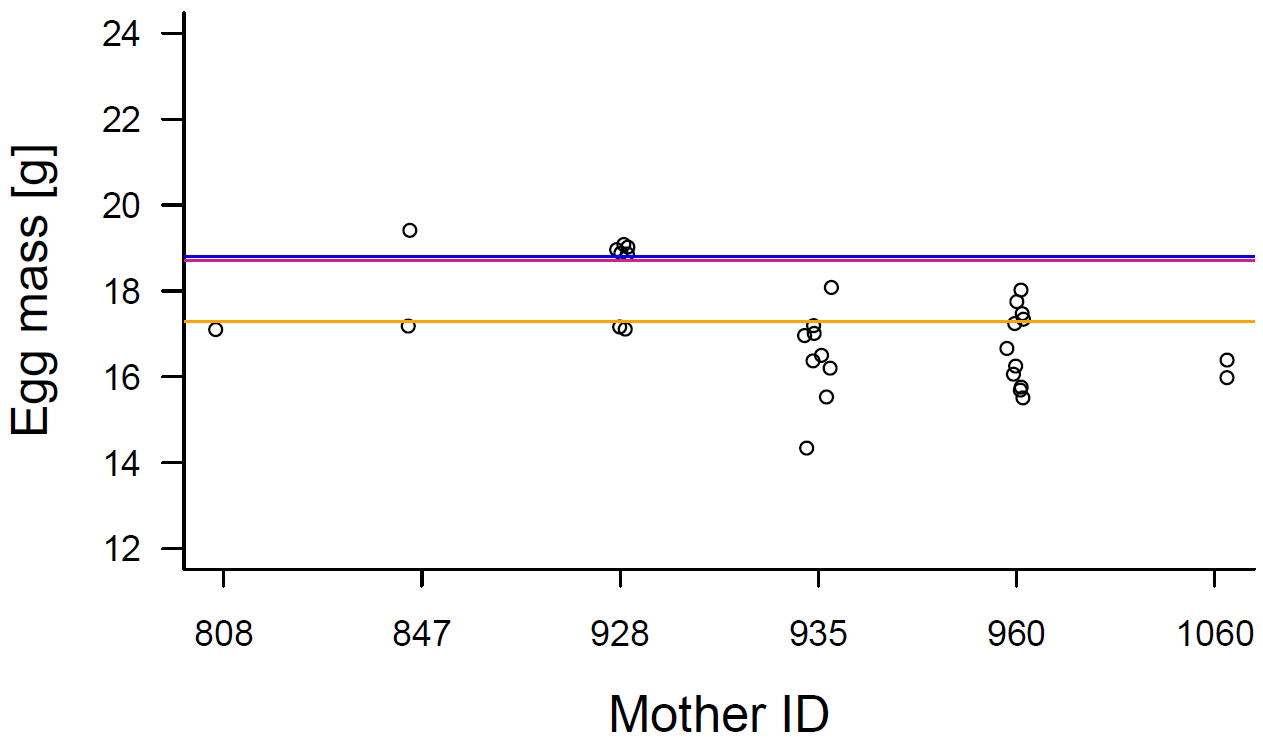


**Supplementary Figure 6 | Egg mass of fertile eggs laid by Faeder females grouped by female ID**. Horizontal lines indicate mean sizes of eggs laid by Independent (N_Eggs_=376, N_Females_=67; blue), Satellite (N_Eggs_=66, N_Females_=17; purple) and Faeders females (N_Eggs_=32, N_Females_=6; orange).

**Supplementary References**

1. Goymann, W. *et al.* Testosterone and corticosterone during the breeding cycle of equatorial and European stonechats (*Saxicola torquata axillaris* and *S. t. rubicola*). *Horm. Behav.* **50**, 779–785 (2006).

2. Goymann, W., East, M. L. & Hofer, H. Androgens and the role of female ‘hyperaggressiveness’ in spotted hyenas (*Crocuta crocuta*). *Horm. Behav.* **39**, 83–92 (2001).

3. Schwabl, H. Yolk is a source of maternal testosterone for developing birds. *Neurobiology* **90**, 11446–11450 (1993).

4. Giraldo-Deck, L. M. *et al.* Development of intraspecific size variation in black coucals, white-browed coucals and ruffs from hatching to fledging. *J. Avian Biol.* **51**, 1–14 (2020).

5. Rahn, H. & Paganelli, C. V. Relationship of avian egg weight to body weight. *Auk* **92**, 750–765 (1975).

6. Carter, K. L. & Kempenaers, B. Eleven polymorphic microsatellite markers for paternity analysis in the pectoral sandpiper, *Calidris melanotos*. *Mol. Ecol. Notes* **7**, 658–660 (2007).

7. Thuman, K. A., Widemo, F. & Piertney, S. B. Characterization of polymorphic microsatellite DNA markers in the ruff (*Philomachus pugnax*). *Mol. Ecol. Notes* **2**, 276–277 (2002).

8. Farrell, L. L., Dawson, D. A., Horsburgh, G. J., Burke, T. & Lank, D. B. Isolation, characterization and predicted genome locations of ruff (*Philomachus pugnax*, AVES) microsatellite loci. *Conserv. Genet. Resour.* **4**, 763–771 (2012).

9. Griffiths, R., Double, M. C., Orr, K. & Dawson, R. J. G. A DNA test to sex most birds. *Mol. Ecol.* **7**, 1071–1075 (1998).

10. Verkuil, Y. I. *et al.* The interplay between habitat availability and population differentiation: A case study on genetic and morphological structure in an inland wader (Charadriiformes). *Biol. J. Linn. Soc.* **106**, 641–656 (2012).

11. Dawson, D. A., Bird, S., Horsburgh, G. J. & Ball, A. D. Autosomal and Z-linked microsatellite markers enhanced for cross-species utility and assessed in a range of birds, including species of conservation concern. *Conserv. Genet. Resour.* **7**, 881–886 (2015).

12. Slate, J., Hale, M. C. & Birkhead, T. R. Simple sequence repeats in zebra finch (*Taeniopygia guttata*) expressed sequence tags: A new resource for evolutionary genetic studies of passerines. *BMC Genomics* **8**, (2007).

13. Klein, Á. *et al.* Microsatellite markers characterized in the barn owl (*Tyto alba*) and of high utility in other owls (Strigiformes: AVES). *Mol. Ecol. Resour.* **9**, 1512–1519 (2009).
