# Supplementary material for: Intralocus conflicts associated with a supergene
Source: Nat Commun. 2022 Mar 16;13:1384. doi: 10.1038/s41467-022-29033-w (PMC8927407; doi:10.1038/s41467-022-29033-w)
Supplement: Supplementary file 3 — Reporting Summary [file 41467_2022_29033_MOESM3_ESM.pdf]

Corresponding author(s): Lina M. Giraldo-Deck, David B. Lank, Clemens Küpper

Last updated by author(s): Dec 15, 2021

Reporting Summary

Nature Portfolio wishes to improve the reproducibility of the work that we publish. This form provides structure for consistency and transparency in reporting. For further information on Nature Portfolio policies, see our Editorial Policies and the Editorial Policy Checklist.

Statistics

For all statistical analyses, confirm that the following items are present in the figure legend, table legend, main text, or Methods section.

n/a	Confirmed	
☐	☒	The exact sample size (n) for each experimental group/condition, given as a discrete number and unit of measurement
☐	☒	A statement on whether measurements were taken from distinct samples or whether the same sample was measured repeatedly
☐	☒	The statistical test(s) used AND whether they are one- or two-sided<br>*Only common tests should be described solely by name; describe more complex techniques in the Methods section.*
☐	☒	A description of all covariates tested
☐	☒	A description of any assumptions or corrections, such as tests of normality and adjustment for multiple comparisons
☐	☒	A full description of the statistical parameters including central tendency (e.g. means) or other basic estimates (e.g. regression coefficient) AND variation (e.g. standard deviation) or associated estimates of uncertainty (e.g. confidence intervals)
☒	☐	For null hypothesis testing, the test statistic (e.g. F, t, r) with confidence intervals, effect sizes, degrees of freedom and P value noted<br>*Give P values as exact values whenever suitable.*
☐	☒	For Bayesian analysis, information on the choice of priors and Markov chain Monte Carlo settings
☐	☒	For hierarchical and complex designs, identification of the appropriate level for tests and full reporting of outcomes
☐	☒	Estimates of effect sizes (e.g. Cohen's d, Pearson's r), indicating how they were calculated

Our web collection on statistics for biologists contains articles on many of the points above.

Software and code

Policy information about availability of computer code

Data collection	Data were collected without the use of any software.
Data analysis	For parentage analyses, alleles were assigned with GeneMapper 4.0. For data analyses we used the functions sim and survfit of the R package arm (version 1.11.1; ref. 62 in manuscript) and survival (version 3.1.12; ref. 64 in manuscript). Both packages were used with R 4.0.0 (ref. 63 in manuscript). The code for the evolutionary model and all statistical analyses are provided as R scripts (https://dx.doi.org/10.17617/3.71).

For manuscripts utilizing custom algorithms or software that are central to the research but not yet described in published literature, software must be made available to editors and reviewers. We strongly encourage code deposition in a community repository (e.g. GitHub). See the Nature Portfolio guidelines for submitting code & software for further information.

Data

Policy information about availability of data

All manuscripts must include a data availability statement. This statement should provide the following information, where applicable:

- Accession codes, unique identifiers, or web links for publicly available datasets
- A description of any restrictions on data availability
- For clinical datasets or third party data, please ensure that the statement adheres to our policy

Raw data and scripts are stored in Edmond the Open Research Data Repository of the Max Planck Society (https://dx.doi.org/10.17617/3.71).

Field-specific reporting

Please select the one below that is the best fit for your research. If you are not sure, read the appropriate sections before making your selection.

☐ Life sciences ☐ Behavioural & social sciences ☒ Ecological, evolutionary & environmental sciences

For a reference copy of the document with all sections, see nature.com/documents/nr-reporting-summary-flat.pdf

Ecological, evolutionary & environmental sciences study design

All studies must disclose on these points even when the disclosure is negative.

Study description

We examined sexual antagonism as a mechanisms of balancing selection maintaining an autosomal inversion polymorphism. We studied a prominent three morph system where an autosomal supergene encodes different male reproductive strategies. The two inversion haplotypes are dominant and homozygous lethal. Because the inversion is autosomal, the three morphs occure in both sexes but little is known how they influence female reproductive success. The lethality of homozygotes also implies substantial fitness costs from matings between inversion morphs, which are particularly borne by the inversion females, who provide all parental care. Females are hence an important but understudied demographic class, whose reproductive investment and success is a key component in the maintenance of the polymorphism in this system.
We investigated variation in female reproductive success, particularly laying rate, egg size and offspring survival in relation to the different supergene variants in a controlled setting. Based on these results, we explored potential mechanisms keeping the inversion polymorphism stable.

Research sample

We studied reproductive success of captive breeding Ruff (Calidris pugnax) females with different supergene variants at Simon Fraser University in Burnaby, British Columbia, Canada. Ruffs feature three well-described morphs that are best described in males as the males differ in aggressive and courtship behaviour, body size, circulating hormone levels, and relative testis size. The male morphs consist of 1) large territorial Independents gather on leks and compete aggressively for visiting females, 2) diminutive Faeders sneak copulations through female mimicry, and 3) semi-cooperative, intermediate-sized Satellites display on leks with Independents. The differences between morphs are encoded by an autosomal inversion region with dominant inversion alleles meaning that females also belong to one of the three morphs. The study population consisted of approximately 300 adult individuals and was established from eggs collected near Oulu, Finland in 1985, 1989 and 1990. It contained individuals from all morphs that have been interbred for multiple generations.

Sampling strategy

In natural ruff populations the inversion morphs are rare (Satellites approx. 16% and Faeders <1%) and ruff females and their chicks are very cryptic during the breeding season. The rarity of these morphs means that required sample size for comparisons of reproductive success are difficult to obtain in the wild. Therefore, we collected data over three years from a captive population that contains females of the inversion morphs at higher frequencies. Based on previous experiences on the reproductive output of the population and logistic constraints, we decided to collect all available data of females and their offspring over three years. We monitored the reproductive investment and success of 186 ruff females (118 Independents, 48 Satellites and 20 Faeders) that laid a total of 962 eggs. All eggs and offspring experienced standardized incubation and rearing conditions. Sample sizes refer to all eggs laid during the study period except those that had to be excluded (details in data exclusion). These sample sizes were sufficient to show statistically clear differences in reproductive output between Faeder and Independent or Satellite females, although further undetected differences may still exist between Satellites and Independents (see discussion).

Data collection

We (authors LMGD, JLL, DBL, CK and students) collected eggs several times per day during daylight hours. We (LMGD, JLL, DBL, CK and students) weighed each egg to the nearest 0.01g, marked them individually and incubated (LMGD) them at 37.5°C and 55% humidity. LMGD candled eggs every four days to confirm the progress of embryonic development. LMGD opened eggs with stalled development and determined the age of the dead embryo based on Hamburger Hamilton embryonic developing stages from chicken (Gallus gallus) that have a similar incubation period. We (LMGD, JLL, DBL, CK and students) individually colour-ringed and hand-raised hatched chicks together with other chicks of similar age in heterosexual and heteromorphic groups under ad libitum food conditions until an age of 20 days when they fledge. Well-being of chicks was recorded at least twice a day by LMGD, JLL, DBL, CK and students. To determine the sex, morph and genetic mother of the offspring with molecular markers, LMGD, JLL or CK took blood or tissue samples.To determine yolk steroid concentrations, LMGD extracted the yolk of eggs without any signs of development (HH index≤1) because hormones start to be metabolized by the embryo already a few hours after incubation starts.

Timing and spatial scale

-Timing: Data collection comprised the entire breeding seasons from 2017 to 2019. It started when the first egg was laid and ended when the last hatched chick reached an age of 20 days (the approximate age of fledging). Data collection occurred during following dates: April 18, 2017 - August 6, 2017; April 12, 2018 - August 3, 2018 and April 22, 2019 - August 6, 2019.
-location: aviaries at campus Simon Fraser University in Burnaby, British Columbia, Canada

Data exclusions

All exclusion criteria were pre-established.
Females were held in breeding pens separated by morph and grouped by age. This setting allowed us to assign the maternal morph for each egg and to control for age effects. Exact maternal ID was known only for the subset of eggs that had developed (N Developed=472 eggs: 374 eggs produced by 67 Independents, 66 eggs produced by 17 Satellites and 32 eggs produced by six Faeders). 72 eggs that had started development were removed from incubation as part of a different experiment.
To analyze egg mass and deviation from expected egg mass, we used all eggs with known mother ID, because egg mass is highly variable between individuals and this data set allowed us to control for individual female variation (Fig. 1). However, for some females we did not have their first year adult body weights. Therefore, only eggs with known body weights of their mothers were used to analyze the deviation from the expected egg mass (Fig. 1b).
Similar for hatching success we used all eggs with known mother ID, but excluded the eggs that had been removed from incubation,

because it was unknown if they would have hatched and we calculated hatching probability using a binomial distribution (Fig. 2). However, we included the eggs that had been removed from incubation as censored data points with unknown fate when we modeled daily survival (Supplementary Fig. S4). For fledging success, we used all hatched chicks (Fig. 2, Supplementary Fig. S5).
To analyze whether female morphs have different laying rates, we used each pen's per capita laying rate as a response variable (i.e. the mean laying rate per female in a pen). The laying rate incorporates all eggs, including those with unknown mother ID. We used this approach because the proportion of infertile eggs differed across morphs, meaning that restricting the analysis to the subset of developed eggs would have biased the results (Supplementary Fig. S1).
To determine steroid hormone allocation in yolks we sampled only eggs without any signs of embryonic development, because hormones start to be metabolized by the embryo already a few hours after incubation starts. For these samples we were unable to obtain embryonic DNA, which would have been necessary to determine the mother ID. Consequently, some of these eggs may have been laid by the same female leading to pseudoreplication. To minimize the impact of pseudoreplication we sampled only eggs belonging to the same pen when they were either laid on the same day, or eggs that were laid 14 days after the last sampling from the same pen, because ruffs typically produce four egg clutches with a minimal egg laying interval of one day (Supplementary Fig. S4).

Reproducibility

To ensure the reproducibility of our findings, we had a controlled set up that was repeated over all study years. The 186 females were assigned to 33 morph- and age-specific breeding pens. Conditions, densities and egg collection regime were similar among pens. All eggs and offspring experienced standardized incubation and rearing conditions. All data were pooled and analysed together. All data and code to reproduce results and figures are made available at acceptance of the manuscript.

Randomization

For this study we determined the morph of each female genetically and then assigned these females randomly to morph and age specific pens (N Independents = 19, N Satellites =11, N Faeders = 3). The setting allowed us to assign the maternal morph for each egg laid (N=962 eggs) and control for confounding effects. Eggs were labelled, weighed and placed into the incubator in the order they were found and chicks were marked and reared in the order they hatched, avoiding systematic clusters of maternal morphs, offspring morphs or offspring sexes inside the incubator or in rearing pens. No further randomization occurred as neither females (nor their offspring) were experimentally manipulated.

Blinding

Technical assistants performed genetic and endocrinal analyses blind to pen & egg information. Handlers of eggs and chicks other than LGD, DBL and CK, were blind to the study goals. However, blinding was not relevant for data collection, because measurements (egg mass and survival) were not subjective.

Did the study involve field work? ☐ Yes ☒ No

Reporting for specific materials, systems and methods

We require information from authors about some types of materials, experimental systems and methods used in many studies. Here, indicate whether each material, system or method listed is relevant to your study. If you are not sure if a list item applies to your research, read the appropriate section before selecting a response.

Materials & experimental systems

n/a	Involved in the study	
☒	☐	Antibodies
☒	☐	Eukaryotic cell lines
☒	☐	Palaeontology and archaeology
☐	☒	Animals and other organisms
☒	☐	Human research participants
☒	☐	Clinical data
☒	☐	Dual use research of concern

Methods

n/a	Involved in the study	
☒	☐	ChIP-seq
☒	☐	Flow cytometry
☒	☐	MRI-based neuroimaging

Animals and other organisms

Policy information about studies involving animals; ARRIVE guidelines recommended for reporting animal research

Laboratory animals

We studied ruff females and their offspring. The females were housed in adjacent pens in an outdoor aviary with unrestricted access to food and water. Females had access to males for at least two hours daily. Collected eggs were incubated at 37.5°C and 55% humidity in automatically turning table top incubators. At 19 – 20 days of incubation, when eggs had clear star pips, they were moved to individual sections of non-turning hatchers, maintained at 37.2°C and 85% humidity. Newly hatched chicks were individually marked and remained in hatchers approx. 12-24 hours post hatch, until they became active and interested in pecking. Chicks were then moved to ca. 0.53m3 brooder boxes maintained at 37.2°C, with ad libitum water and food conditions. Heterosexual and heteromorphic groups of up to eight chicks were held together as randomly formed 'broods' of chicks hatching at the same time. As chicks reached 25-30 g and began to thermoregulate, they were moved to a run at ca. 21°C with access to a heat lamp. At an age of 17 days, they were moved to a pen in the outside aviary and after the breeding season juveniles were pooled together with adults.

Wild animals

no wild animals were used in this study

Field-collected samples

no field collected samples were used in this study

Ethics oversight

Housing and rearing (permit #1232B-17) were approved by the Animal Care Committee of Simon Fraser University operating under guidelines from the Canadian Council on Animal Care.

Note that full information on the approval of the study protocol must also be provided in the manuscript.
